# Displacement Sensor Based on a Small U-Shaped Single-Mode Fiber

**DOI:** 10.3390/s19112531

**Published:** 2019-06-03

**Authors:** Chuanxin Teng, Fangda Yu, Shijie Deng, Houquan Liu, Libo Yuan, Jie Zheng, Hongchang Deng

**Affiliations:** 1 Photonics Research Centre, Guilin University of Electronic Technology, Guilin 541004, China; shijie.deng@guet.edu.cn (S.D.); liuhouq@guet.edu.cn (H.L.); lbyuan@guet.edu.cn (L.Y.); 2 Guangxi Key Laboratory of Automatic Detection Technology and Instrument Foundation (No.), Guilin 541004, China; 3 State Key Laboratory on Integrated Optoelectronics, College of Electronic Science and Engineering, Jilin University, Changchun 130012, China; yufd15@mails.jlu.edu.cn (F.Y.); zhengjie@jlu.edu.cn (J.Z.)

**Keywords:** displacement measurement, bent fiber interferometer, a small U-shape

## Abstract

A simple structure and easily fabricated displacement sensor was proposed and demonstrated based on a bending-induced fiber interferometer. In the design, the fiber interferometer was formed only by bending the single-mode fiber into a small U-shape without splicing, tapering, or heating pre-processing, which effectively reduces the complexity of the fabrication process, greatly enhances the mechanical strength of the sensor, and lowers the cost in the displacement sensing applications. The displacement sensing performances for the sensor with different bending radii of 3.3 mm, 4.4 mm, 5.0 mm, and 6.3 mm were investigated. Experimental results showed that the sensor had a good linear response, and for the bending radii of 3.3, 4.4, 5.0, and 6.3 mm, the proposed sensors showed high sensitivities of 134.3, 105.1, 120.9, and 144.1 pm/μm, respectively.

## 1. Introduction

Displacement sensors play an important role in many applications, such as structural health monitoring, industrial control, and so on. The measurements of many physical parameters like stress, strain, temperature, and acceleration could be converted to the measurement of displacement. Thanks to the development of optical fiber technology, many types of optical-fiber-based sensors have been proposed for detecting the displacement. Compared with the electronic counterparts, the optical fiber displacement sensors have the advantages of simple structure, electromagnetic immunity, and the ability for remote sensing and real-time sensing. Generally, the reported optical-fiber-based displacement sensors are mainly divided into two types: intensity-modulated [1,2] and wavelength-modulated sensors [3]. The intensity-modulated displacement sensors are low cost; however, their accuracy is often low due to the influence of the light source fluctuations, and compensation techniques are required to improve the accuracy. In contrast, the wavelength-modulated sensors detect the wavelength shift, which have a better accuracy. In recent years, many kinds of wavelength-modulated displacement sensors have been reported, such as the fiber Bragg grating (FBG)-based displacement sensors [4], the long-period grating (LPG)-based displacement sensors [5], the optical fiber surface plasmon resonance (SPR)-based displacement sensors [6], and the photonic crystal fiber based displacement sensors [7]. Those sensors, however, either need complex fabrication process including the grating inscription or the gold plating, or using the special optical fibers, which are time consuming and expensive, restricting their development in practical applications.

In recent years, many fiber-based interferometers have been proposed for displacement sensing, which are simple structures, easily fabricated, and low-cost. In 2011, Q. Wu et al. proposed a simple single-mode/multimode/single-mode (SMS) fiber structure for displacement sensing, but its sensitivity was only 5.89 pm/μm [8]. Recently, K. Tian et al. established a balloon-shaped bent multimode fiber structure for displacement measurement, where the sensitivity was raised to 36 pm/μm over the displacement range of 0–100 μm [9]. In 2012, H. Luo et al. employed a bent microfiber taper for displacement measurement, and a high sensitivity of 102 pm/μm was achieved in the displacement range of 0–30 μm, but the microfiber was very fragile [10]. In addition, J. Chen et al. proposed a bent core-offset fiber Mach-Zehnder interferometer (MZI) for displacement sensing, where its sensitivity was 227 pm/μm in the displacement range of 0–1000 μm [11]. The reported bent-fibers interferometers above, however, also need some special treatments in order to make the core mode couple to the cladding mode and recouple back; for example, the arc fusion splicer was used when fabricating the SMS fiber structure or the core-offset structure, and the flame-heated treatment was implemented when fabricating the microfiber. While on the other hand, the fiber interferometers could be formed by only bending the single-mode fiber. Y. Fang et al. [12] and X. Zhang et al. [13] proposed the bent optical fibers for refractive index (RI) sensing. This bending-induced interference could also be used for displacement sensing. In 2013, L. Xu et al. reported a highly-sensitive semicircular-fiber-structured displacement sensor, but the sensor was also prepared using a flame-heated process, which made it difficult to fabricate the structure precisely, and the measurement range was limited after the heating process [14].

In this paper, we propose and demonstrate a small U-shaped optical fiber displacement sensor. It does not need any special treatments including splicing, tapering, or flame heating, and it can be constructed by just removing the coating layer of the fiber and bending it. By bending the bare standard single-mode fiber into a suitable bending radius, an MZI could be constituted. The established bending-induced interference was analyzed theoretically, and the displacement sensing performance was investigated experimentally. Experimental results showed that the proposed sensor had a comparable high sensitivity to the displacement. Benefiting from its excellent advantages of simple configuration, cost-effective, easy fabrication, and better mechanical strength, this displacement sensor could be a competitive candidate for accurate displacement measurement in practical applications.

## 2. Device Structure and Operating Principle

**Figure 1:**
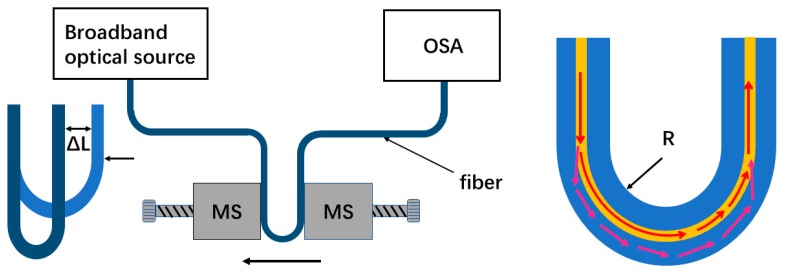
The schematic of the experimental setup and the bending fiber structure.

Figure 1 shows the schematic illustration of an experimental setup using a bent single mode fiber (G.652D, YOFC, Wuhan, China) for displacement sensing. The single mode fiber was placed and bent between two translation stages (MS), and the fiber coating layer of the bending section was removed. The ultraviolet (UV) glue was used to immobilize the single mode fiber to the stages. For measuring the displacement, one stage was kept fixed and the other was moved toward it (with a 10 μm displacement step) to apply an incremental displacement to the bending fiber. The bending radius of the single mode fiber was defined as *R*. A broadband light source (ASE) (A-0002, HOYATEC, Shenzhen, China) was connected into the fiber, and an optical spectrum analyzer (OSA) (AQ6370D, YOKOGAWA, Tokyo, Japan) with a highest spectral resolution of 0.02 nm was used to record the transmission spectrum.

**Figure 2:**
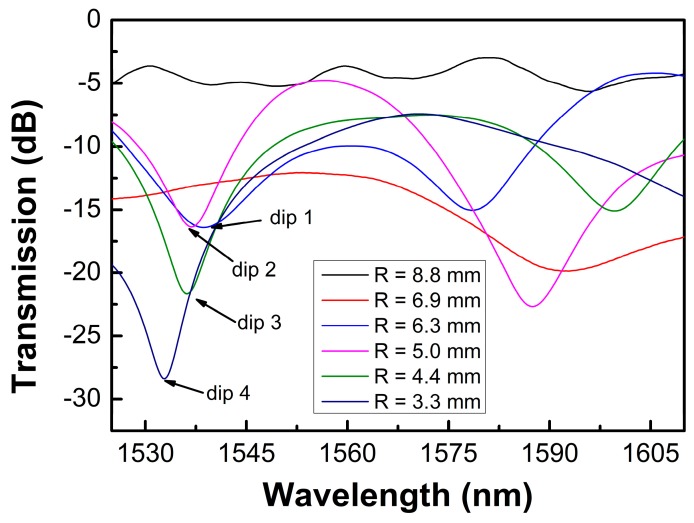
Transmission spectra of the bending fiber with different bending radii.

As shown in Figure 1, when the light passes through the bending section, part of the light is coupled to some cladding modes, and after both the light in the core mode and the cladding modes propagating along the bending section to the bending end, the cladding modes are partly re-coupled back into the core and interfere with the rest of the core mode due to the accumulated phase difference between them. Therefore, the different optical paths of the core mode and cladding modes form an intermodal interferometer. Figure 2 shows the transmission spectra of the bending fiber with different bending radii in the wavelength range of 1525–1610 nm. It is noted that when the bending radius was larger than 8.8 mm, the interference fringes were not obvious, but as the bending radius decreased, they became more visible. This may be because the power of the cladding modes was low when the bending radius was large. While for the small U-shaped fibers with the bending radius range of 6.3–3.3 mm, the situation was changed, and the extinction ratios of the interference could reach to 10–20 dB, which were available for the displacement measurement.

The transmission spectrum of the interferometer is simply described as a two-mode interference [15],

(1) $$I=I_{\mathrm{co}}+I_{\mathrm{cl}}+2\sqrt{I_{\mathrm{co}}I_{\mathrm{cl}}}\cos\phi$$

where *I* is the light intensity of the output light, and *I*_co_ and *I*_cl_ are the intensities of the core mode and cladding mode, respectively. ϕ is the phase difference of the two interference modes, which can be expressed as [16]:

(2) $$\phi =\frac{2\pi \Delta n_{eff}L}{\lambda }$$

where $\Delta n_{eff}$ is the effective refractive index (RI) difference between the core mode and cladding mode; for a small bending radius, the effective refractive index of the core mode is lower than those of the cladding modes, therefore, $\Delta n_{eff}=n_{cl}-n_{co}$. L=πR is the arm length of the interferometer, and λ is the operating wavelength in a vacuum. When ϕ=(2i+1)π, *i* is an integer, the interference dip will appear at specific wavelengths, which can be expressed as [17]:

(3) $$\lambda_{i}=\frac{2\Delta n_{eff}L}{2i+1}$$

When the displacement increases ΔL (right stage moving to the left), as shown in Figure 1, the bending radius decreases and the according arm length of the interferometer becomes $L^{\prime }=\left(R-\Delta L/2\right)\pi$, which approximately equals *L* when the displacement ΔL is small. On the other hand, as the bending radius decreases, the effective RI of the cladding mode will increase, while that of the core mode will remain constant [18]. Therefore, the dip wavelength shift could be expressed as:

(4) $$\delta \lambda_{i}=\frac{2\left(\Delta n_{eff}+\delta n\right)L}{2i+1}-\frac{2\Delta n_{eff}L}{2i+1}=\frac{2\delta nL}{2i+1}$$

where *δn* is the change of effective RI difference caused by the increased displacement. Thus, the transmission spectrum dips exhibit a red shift as the displacement increases.

**Figure 3:**
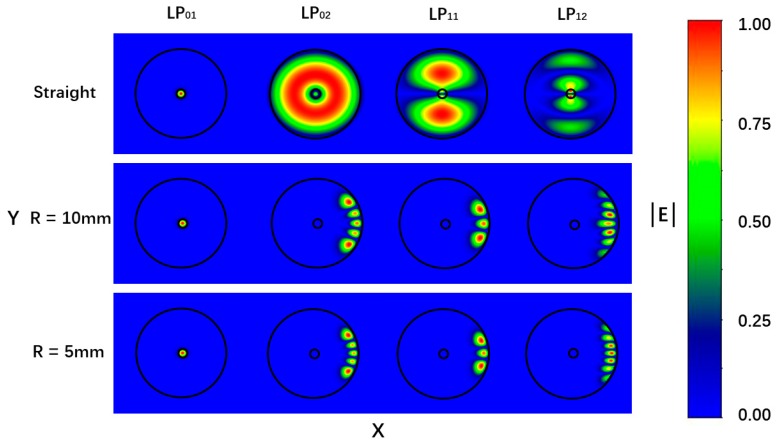
Mode fields of the first two orders of the LP_0n_ and LP_1n_ for the straight fiber and bending fibers with the bending radius of 10 and 5 mm, respectively.

**Figure 4:**
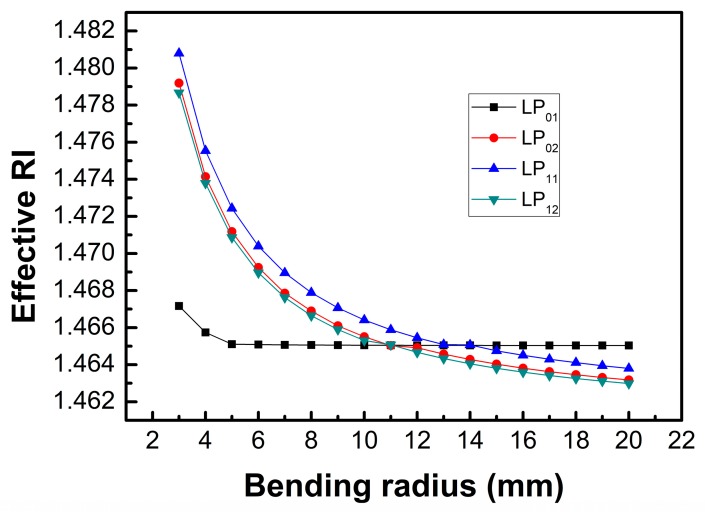
The effective RIs of the first two orders of the LP_0n_ and LP_1n_ modes with different bending radii.

In addition, the bend will eliminate the random variation effect of the fiber polarization state and makes the fiber with a polarization-maintaining function. The numerical simulations of the transverse mode profiles for the bending fibers have been performed by using an OptiBPM software (version 10.0). The numerical mesh size in the profile directions of X and Y were 0.8 μm, and the diameter and RI for the fiber core and cladding were 8.2/125 μm and 1.4682/1.4628, respectively. Figure 3 shows the mode fields of the first two orders of the LP_0n_ and LP_1n_ modes for the straight fiber and bending fibers with the bending radii of 10 and 5 mm, respectively. For the bending case, the center of fiber curvature was located to the left of the figure, and the core-cladding and cladding-air boundaries were indicated by the circular outlines. It was found that the mode field distributions were symmetric for the straight fiber; however, they tended to distort and shift away from the center of the curvature for the bending fiber. It was also found that the distortions of the mode fields were more obvious for the fiber with the bending radius of 5 mm than that with the bending radius of 10 mm. Figure 4 shows the effective RIs for the core mode and the cladding modes with different bending radius. It was found that the effective RIs of the cladding modes became larger than that of the core mode when the bending radius was smaller than 10 mm, and as the bending radius decreased, the effective RIs of the cladding modes increased, while that of the core mode increased only when the bending radius was smaller than 5 mm. 

## 3. Experimental Results and Discussions

**Figure 5:**
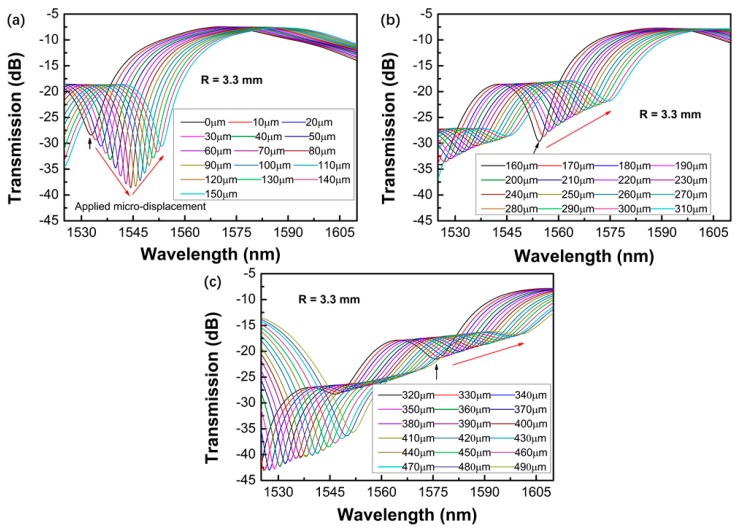
Transmission spectra of the proposed sensors varied with the displacement ranges of 0–150 μm (**a**), 160–310 μm (**b**), and 320–490 μm (**c**) for the bending radius of 3.3 mm.

**Figure 6:**
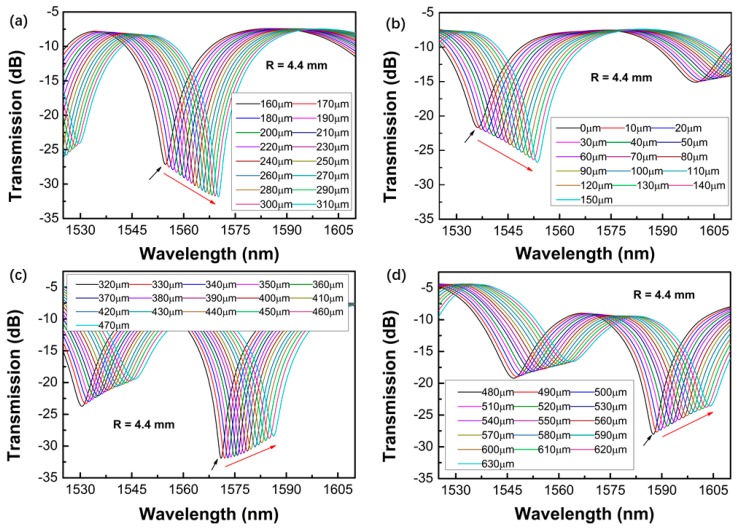
Transmission spectra of the proposed sensors varied with the displacement ranges of 0–150 μm (**a**), 160–310 μm (**b**), 320–470 μm (**c**), and 480–630 μm (**d**) for the bending radius of 4.4 mm.

**Figure 7:**
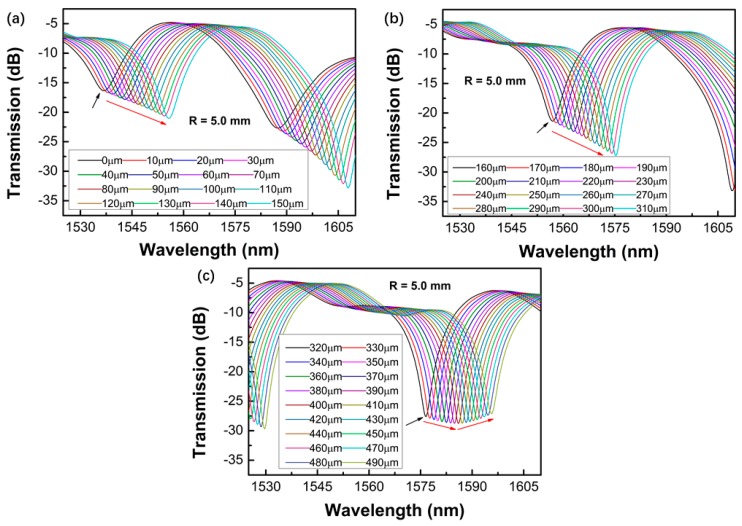
Transmission spectra of the proposed sensors varied with the displacement ranges of 0–150 μm (**a**), 160–310 μm (**b**), and 320–490 μm (**c**) for the bending radius of 5.0 mm.

**Figure 8:**
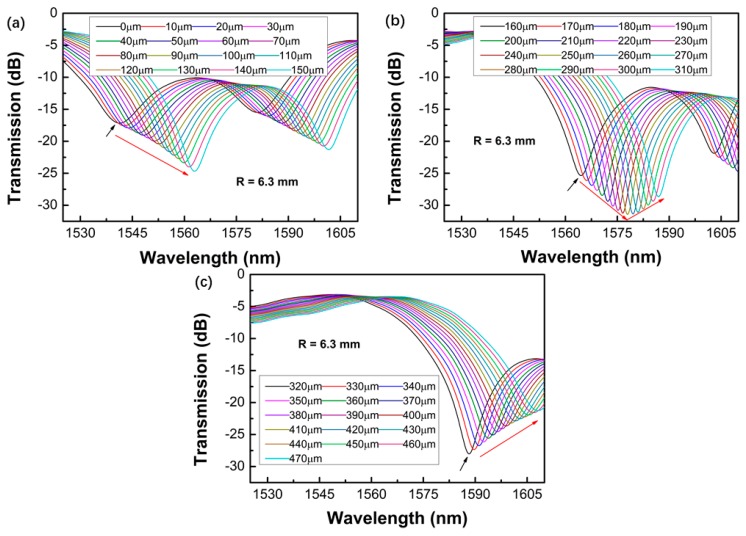
Transmission spectra of the proposed sensors varied with the displacement ranges of 0–150 μm (**a**), 160–310 μm (**b**), and 320–470 μm (**c**) for the bending radius of 6.3 mm.

Figure 5, Figure 6, Figure 7 and Figure 8 shows the transmission spectra of the proposed sensors when they were subjected to an increased displacement with a step of 10 μm. The certain displacement value ∆*L* was related to the bending radius, as well as the interferometer arm lengths *L*. Therefore, the proposed sensors with different bending radii were fabricated, and their displacement sensing performances were studied. The experiment was performed at room temperature. Figure 5, Figure 6, Figure 7 and Figure 8 shows the contrastive interference patterns of sensors with different bending radii of 3.3, 4.4, 5.0, and 6.3 mm, respectively. Here, we defined the initial displacement as 0 μm. It was found that the interference patterns shifted to the longer wavelength as the displacement increased, as expected based on theoretical data. It was also found that all the spectra dips increased first and then decreased, which may be because the power of the dominant cladding mode that couples with the core mode increased first and decreased later as the displacement increased. 

**Figure 9:**
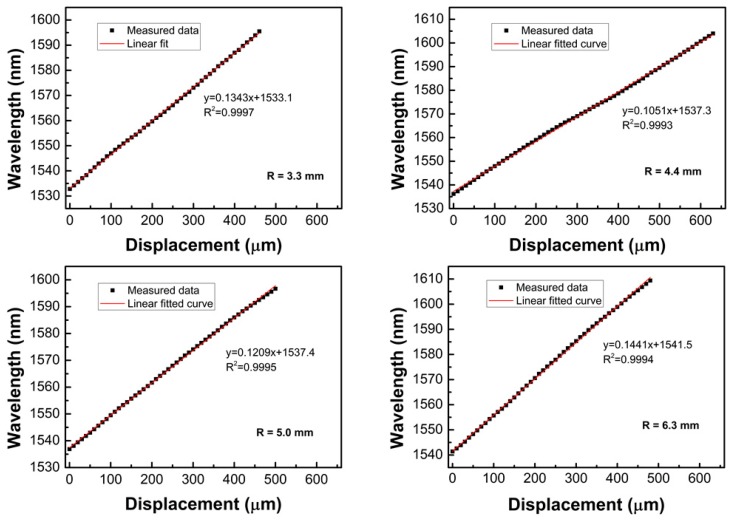
Resonant wavelength dips as a function of displacement for the sensors with different bending radii of 3.3, 4.4, 5.0, and 6.3 mm, respectively.

Figure 9 shows the wavelength changes of dip 1–4 (marked in Figure 2) as a function of the displacement. It was found that the sensor had a good linear response in the measured displacement range. The sensitivities were defined as the slopes of the fitting curves, which were 134.3, 105.1, 120.9, and 144.1 pm/μm for the sensors with a bending radius of 3.3, 4.4, 5.0, and 6.3 mm, respectively. The obtained sensitivities were much higher than those of FBG-based displacement sensors [4]. The standard deviations of the measured data were also evaluated, where five data were recorded for every measured point, and the maximum standard deviation was about 0.0837. It was also found that a narrower free spectral range (FSR) could be obtained with a smaller bending radius, where the FSR is approximated as FSR = *λ*^2^/Δ*n_eff_L*; however, if the bending radius was too small, the transmission loss was high and the extinction ratio of the transmission spectrum decreased. Similarly, if the bending radius of the fiber was less than 3 mm, the fiber was easily damaged, which made it unsuitable for displacement sensing applications.

**Figure 10:**
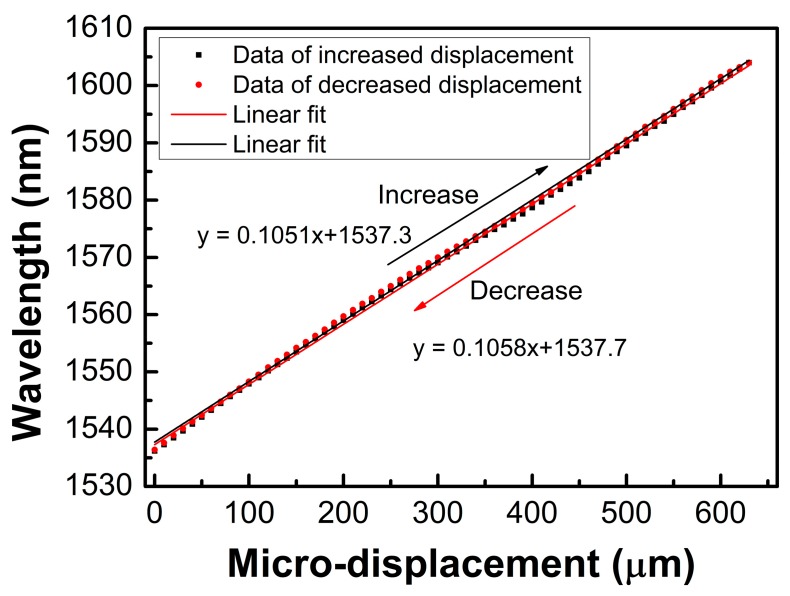
Spectrum variation as a function of displacement including displacement increase and decrease processes.

We also performed a reversed cycled measurement for the sensor with a bending radius of 4.4 mm, where the displacement was increased from 0 μm to 630 μm and decreased from 630 μm back to 0 μm with a step size of 10 μm. It was found that the dip wavelength shifted to the shorter wavelengths when the displacement decreased. The results are shown in Figure 10. It can be found that the sensitivities of wavelength dip 3 for the increased and decreased displacement processes were 105.1 and 105.8 pm/μm with a linear regression coefficient (R^2^) of 0.9993 and 0.9992, respectively, which indicates that there was no hysteresis present and the repeatability of the sensor was good. A comparison between the proposed sensor in this work and the other sensors cited in this article is listed in Table 1. It is clear that the achieved displacement sensitivities of the sensor in this work compare favorably with other sensors, and the proposed sensor has a large sensing range. In addition, this proposed sensor has a simple structure and easy fabrication, which can be used in accurate displacement measurement fields.

## 4. Conclusions

We have presented a displacement sensor based on a bending single-mode fiber. The sensor was fabricated with a simple structure and was easily fabricated just by removing the coating layer of the fiber and bending it into a small U-shape. There were no special treatments in the fabrication process like splicing, tapering, or heating, which enhanced the mechanical strength of the sensor. The sensing performances of the sensors with different bending radii were investigated experimentally. Experimental results showed that the sensor had a good linear response, and the highest sensitivity obtained was up to 144.1 pm/μm in a large displacement variation range of 470 μm. The proposed sensor also provided an experimentally proven good repeatability, and it is a low-cost solution for displacement measurement. 

## Figures and Tables

**Table 1:** Comparison of the performance of different displacement sensors.

Measurement Configuration	Displacement Sensitivity (pm/μm)	Measurement Range (μm)	Special Treatment	Ref.
FBG	0.567	0–3000	Grating inscribing	[4]
LPFG	216	0–140	Grating inscribing	[5]
MMI	36	0–100	Splicing	[9]
Bent microfiber	102	0–30	Tapering	[10]
MZI	227	0–1000	Splicing	[11]
Semicircular fiber	1100	0–80	Heating	[14]
**Proposed sensor**	**144**	**0–470**	**None**	**This work**
